# Education and lifestyle predict change in dietary patterns and diet quality of adults 55 years and over

**DOI:** 10.1186/s12937-019-0495-6

**Published:** 2019-11-07

**Authors:** Maree G. Thorpe, Catherine M. Milte, David Crawford, Sarah A. McNaughton

**Affiliations:** Institute for Physical Activity and Nutrition (IPAN), School of Exercise and Nutrition Sciences, Deakin University, Geelong, Australia

**Keywords:** Diet quality, Principal component analysis, Dietary patterns, Older adults, Predictors of change, Cohort

## Abstract

**Background:**

Diet is a key risk factor for chronic disease, and an increasing concern among older adults. We aim to examine the changes in dietary patterns using principal component analysis and a diet quality index among older adults and examine the predictors of dietary change over a 4 year period.

**Methods:**

Data was obtained via a postal survey in a prospective cohort, the Wellbeing Eating and Exercise for a Long Life (WELL) study. Australian adults aged 55 years and over (*n* = 1005 men and *n* = 1106 women) completed a food frequency at three time points and provided self-reported personal characteristics. Principal component analysis was used to assess dietary patterns and diet quality was assessed using the 2013 Revised Dietary Guideline Index. The relationships between predictors and change in dietary patterns were assessed by multiple linear regression.

**Results:**

Two dietary patterns were consistently identified in men and women at three time points over 4 years. One was characterised by vegetables, fruit and white meat, and the other was characterised by red and processed meat and processed foods. Reduced consumption of key food groups within the principal component analysis-determined dietary patterns was observed. An increase in diet quality over 4 years was observed in men only. Reported higher education levels and favourable lifestyle characteristics, including not smoking and physical activity, at baseline predicted an increase in healthier dietary patterns over 4 years.

**Conclusions:**

There was stability in the main dietary patterns identified over time, however participants reported an overall decrease in the frequency of consumption of key food groups. Compliance with the Australian Dietary Guidelines remained poor and therefore targeting this population in nutritional initiatives is important. Design of nutrition promotion for older adults need to consider those with lower socioeconomic status, as having a lower level of education was a predictor of poorer dietary patterns. It is important to consider how nutrition behaviours can be targeted alongside other lifestyle behaviours, such as smoking and inadequate physical activity to improve health.

## Background

Diet is an important risk factor for chronic diseases such as cardiovascular disease and type 2 diabetes [1]. Chronic disease is a major health concern among older adults [2] and of particular concern are those within the peri-retirement age group where these conditions begin to manifest. This is also a period of major life transition, with experiences such as changed work-loads, marital transitions, and children leaving home occurring. Transitional life stages may impact health behaviours including diet [3–5], and may provide a window of opportunity to promote dietary change [6]. Improving diet within this age group will help to reduce the burden of disease and improve quality of life in older adults [7].

The importance of foods and overall dietary patterns, rather than single nutrients, has emerged in the last few decades of nutrition research [8]. Exploration of dietary patterns allows the complex nature of diet to be acknowledges by considering the synergistic effects of foods and nutrients within the body as well as considering the way we consume food [9]. There are multiple methods for assessing dietary patterns, namely empirical methods that use statistical techniques to explore the dietary intake data, such as principal component analysis, and theoretically-based dietary patterns based on predefined patterns constructed from hypotheses and scientific evidence regarding nutritional health, such as dietary guidelines and recommendations [10]. Applying multiple methods within the same data set may increase our understanding of the use of these methods and the implication of using different methodologies [11].

Epidemiological studies often assess dietary patterns at just one-time point to represent typical or long-term dietary patterns, however, dietary patterns may change over time particularly during transitional life stages such as retirement. There is little research available that describes the dietary patterns of those within a peri-retirement life stage [12, 13], as many studies of older adults focus on a broader age range or more specifically on the elderly [14, 15]. For example, those with higher occupation and education levels are more likely to be associated with improved diet in adults aged 25–75 years [16] and 50–69 years old [17]. The number of longitudinal studies of dietary patterns in peri-retirement age are limited [17, 18] with only two studies in an Australian population [16, 19]. Furthermore, few existing studies have examined predictors of change in dietary patterns among peri-retirement [17, 18, 20]. Those studies found that a higher level of education and social class predicted individuals continuing with a healthier dietary pattern or improving their dietary pattern over time. In the broader adult population, older age, being female and being of higher socioeconomic position have consistently been shown to be predictors of improved dietary patterns [16, 21–25]. Understanding what predicts change in dietary patterns will inform future research and nutrition promotion interventions. The purpose of this study was to examine the dietary patterns among peri-retirement aged older adults over 4 years using principal component analysis (PCA) and the 2013 Revised Dietary Guideline Index (DGI-2013), and to examine predictors of change in dietary patterns.

## Methods

This study used data collected in the WELL study. The methods of the WELL study have been described in detail elsewhere [26]. In brief, a stratified random sample was selected from the Australian Electoral Commission’s electoral roll, a compulsory register for Australians. The sample was stratified by gender and three levels of Socioeconomic Position (SEP) according to the Index of Relative Socio-economic Disadvantage, a Socioeconomic Index for Areas (SEIFA) score, assigned by the Australian Bureau of Statistics [27]. Fourteen postcodes from three SEIFA categories (low, medium and high SEIFA) and in both rural and urban areas were randomly selected. From these, 134 participants (equal men and women) were randomly selected for the study, resulting in the final sampling pool of 11,256 Australian adults (55–65 years). Those who agreed to participate completed a postal questionnaire in 2010. In 2012 and 2014, participants who had not withdrawn were re-contacted via the post and invited to complete a follow-up survey. (WELL study participant recruitment flow diagram provided in additional file 1). The studies methods were approved by the Deakin University Human Research Ethics Committee (2009–105).

### Dietary intake

Dietary intake at each time point was assessed using a 111-item Food Frequency Questionnaire (FFQ) adapted from the 1995 Australian National Nutrition Survey [28, 29]. The FFQ assessed participant’s dietary intake over the previous 6 months, with nine response categories for each item, ranging from ‘never or less than once a month’ to ‘6+ times per day’. No information was gathered on portion sizes. Data from participants with > 10% of the FFQ data missing were considered invalid [30] and not included in this study while all other missing FFQ responses were considered not consumed [30]. Additional food-behaviour questions concerning daily fruit and vegetable consumption, salt use, trimming the fat from meat and type of bread and milk consumed were also included in the questionnaire. These questions have been evaluated and shown to be valid measures of food intake behaviours [30–35].

### Dietary patterns assessed using principal component analysis (PCA)

Dietary patterns were determined using PCA which has been described in detail elsewhere [11] and the results across all time points have been included in additional file 2. The FFQ items were converted to daily equivalents and categorised into 52 food groups according to nutritional content, culinary usage and food groups in the 2013 Australian Dietary Guidelines (ADG) [36] (Additional file 3). FFQ items consumed (≥ 1 time per week) by less than 10% of the population were combined with other food items where possible or omitted. Only soy beverages were omitted since a large proportion of the sample (91%) indicated they never consumed this item. The daily intake frequencies were used to determine dietary patterns by PCA. Servings per day (frequency), is routinely used to determine empirical dietary patterns [37].

Initially, PCA was conducted separately for each time point to assess whether similar patterns were present over time. The factor loadings for the dietary patterns identified at each time were examined qualitatively and quantitatively using Tucker’s coefficient of congruence [38] to determine the similarity of dietary patterns between time points. Only the patterns that were present at all three time points were included in the analysis. Factor scores for the retained patterns were calculated using the 2010 factor loadings for key foods (factor loadings of | ≥ 0.20|) as the sum of key food group frequencies multiplied by factor loading at each time point. Factor loadings from 2010 were used to calculate factor scores at all three time points for a consistent dietary pattern measure with changes due to frequency of consumption.

### Dietary patterns assessed using diet quality

A diet quality index, the revised Dietary Guideline Index (DGI-2013) [13] was used to assess diet quality, according to the ADG [36] using data from the FFQ and additional food-behaviour questions. A detailed description of the DGI-2013 has been provided elsewhere [13]. The DGI-2013 includes 13 components, each scored out of ten. The total diet quality score range is 0 to 130. There are two categories of components, firstly those that reflect adequate intake of nutritious foods. This includes diet variety, vegetables, fruit, cereal, protein, dairy and water. Secondly those that reflect moderation or limited intake of foods and drinks high in saturated fat and/or added sugar, salt or alcohol and low in fiber. This includes discretionary food, saturated fat, salt, sugar and alcohol consumption. A higher DGI-2013 score indicates greater adherence to the ADG and therefore greater diet quality.

The original DGI has been associated with independently measured daily nutrient intakes, including an inverse relationship with fat and energy consumption and a positive relationship with dietary fibre and several important micronutrients such as β-carotene, vitamin C, folate, calcium, and iron [39]. A higher DGI score has been associated with a higher level of SEP and reduced cardiometabolic risk including Type 2 diabetes, hypertension, obesity and demonstrated reduced weight gain [40–43]. Since being updated, the DGI-2013 has shown similar qualities, with a higher score associated with higher education, being a non-smoker, higher physical activity and lower BMI [13] as well as better health-related quality of life [7].

### Predictors

Potential predictors of dietary change were self-reported in the questionnaires, including socio-demographic characteristics (sex, education, retirement status and relationship status) and health-related behaviours and characteristics (anthropometry, weight loss intentions, smoking status, physical activity and diagnosed cardiometabolic disease-related conditions). Education was collapsed into three categories (no formal qualifications and up to year 10; year 12, trade, apprenticeship, certificate or diploma; and a university degree or higher). Retirement status at baseline was dichotomously coded as yes or no. Relationship status was collapsed from five categories into three (living as married-registered or de facto; separated or divorced; and never married). Baseline (2010) Body Mass Index (BMI) was calculated from self-reported height and weight and categorised according to the following criteria: underweight: BMI < 18.5; healthy: BMI ≥ 18.5 to < 25 kg/m^2^; overweight: BMI ≥ 25 to < 30 kg/m^2^; obese: BMI ≥ 30 kg/m^2^. Few participants were diagnosed with any cardiometabolic disease-related conditions after 2010, therefore the data from the three time points were converted into dichotomous variables; indicating whether they had ever been diagnosed with a cardiovascular (CVD)-related condition (stroke, blood clot, high blood pressure or heart disease) and whether they had ever been diagnosed with a diabetes-related condition (diabetes or impaired glucose tolerance). Weight loss intentions at baseline were categorised into three categories (trying to avoid gaining weight, trying to lose weight and not trying anything or trying to gain weight, since few (< 1%) participants reported trying to gain weight). A collapsed baseline smoking status variable was used (current smoker-occasionally or regularly; former smoker; and never smoked). Self-reported physical activity in the 7 days prior to the questionnaire was assessed using the long version of the International Physical Activity Questionnaire (IPAQ) [44]. IPAQ records the frequency, intensity, and duration of physical activity during the previous week. Only time spent in leisure time physical activity (walking, moderate and vigorous) was considered as research has shown leisure time physical activity is a better predictor of good self-rated health in comparison to work related physical activity [45, 46]. This is also a behaviour that lifestyle interventions may be more successful in addressing. Minutes of leisure time activity per week were calculated by summing the number minutes of moderate intensity leisure time physical activity per week and twice the number of minutes per week spent participating in leisure time vigorous intensity physical activity per week [44]. Participants were classified as to whether they did or did not meet the physical activity recommendations of at least 150 min of activity per week [47]. Age has previously been shown to predict diet [48] and change in diet [16], however, given the narrow age range in the WELL study, it was not explored as a predictor but included as a covariate. Given the known associations with diet; BMI smoking and physical activity [16, 21–25] were also adjusted for in the analysis of predictors were appropriate.

### Statistical analysis

All analyses were stratified by sex since dietary patterns and diet quality differ between men and women [37, 49]. Mixed-effect multi-linear regression with random intercepts for individuals and robust standard errors for the sampling locations (postcodes) was used to assess change in dietary scores across 2010, 2012 and 2014, where the linear effect of time was the predictor. This method recognises the relationship between successive observations while accounting for the individuals’ random effects [50]. Multiple linear regression with robust standard errors for the sampling location (postcodes) was used to assess the relationships between predictors and the change in dietary pattern scores from 2010 to 2014. The outcome (difference between 2010 and 2014) was achieved by using the 2014 score as the outcome adjusting for the baseline (2010) score. This is the recommended method in behavioural science to overcome measurement errors in studies of change [51]. Analysis of the base model was conducted followed by a model adjusting for baseline age, BMI, smoking and physical activity given their association with diet. In the models where these confounders were used as the predictor they themselves were not included in the model as a cofounder. Assumptions for linear regression were tested and adequately met for the models tested.

## Results

### Participant characteristics

In 2010, 4082 questionnaires were returned, 2757 were returned in 2012 and 2542 questionnaires were returned in 2014. Participants were excluded from the analysis if they did not provide their date of birth or sufficient dietary intake data (missing > 10% of FFQ or who were missing additional diet behaviour questions) resulting in the following sample sizes used to determine dietary patterns; 2010: *n* = 1888 men and 2071 women; 2012: *n* = 1269 men and 1428 women and 2014: *n* = 1183 men and 1309 women. For analysis of predictors, only those with complete data at baseline and the final time point were included (*n* = 1005 men and *n* = 1106 women). Participant characteristics are summarised in Table 1.

**Table 1:** Characteristics of Wellbeing Eating and Exercise for a Long Life study participants^a^

Participant characteristics	Men (*n* = 1005)	Women (*n* = 1106)	*P*-Value
Mean age (SD) years	60 (3.0)	60 (3.1)	0.595
Education (%)			
No formal qualifications and up to year 10	29	36	< 0.001
Year 12, trade/apprenticeship or certificate/diploma	39	32	
University degree and higher	32	32	
Retired (%)			
Retired	33	45	< 0.001
Not retired	67	55	
Relationship status (%)			
Living as married	85	77	< 0.001
Separated, divorced or widowed	10	19	
Never married	5	4	
Weight loss intentions (%)			
Not trying anything for my weight	48	34	< 0.001
Trying to avoid gaining weight	26	32	
Trying to lose weight	26	34	
BMI category (%)			
Healthy (BMI ≥ 18.5 to < 25 kg/m^2^)	31	46	< 0.001
Overweight (BMI ≥ 25 to < 30 kg/m^2^)	47	32	
Obese (BMI ≥ 30 kg/m^2^)	22	22	
Smoking status (%)			
Never smoked	46	58	< 0.001
Former smoker	43	34	
Current smoker	11	8	
Meeting physical activity guidelines (%)			
Yes	52	57	0.018
No	48	43	
Diagnosis of CM-related condition (%)			
No CM-related conditions	38	44	0.013
Diagnosed with one or more prior to 2010	49	45	
Newly diagnosed with one or more within 2010–2014	13	11	

Abbreviations: *SD* Standard Deviation, *BMI* Body Mass Indexm, *CM* cardiometabolic

^a^ Baseline (2010) characteristics unless otherwise indicated

^b^ *P* value for difference between gender, *P* < 0.05 is considered significant

### Dietary patterns

#### PCA-derived dietary patterns

In men, four dietary patterns were identified in 2010 [11], four in 2012, and two patterns were identified in 2014. Only two dietary patterns remained consistent across all time points and were therefore retained for further analysis (Table 2). Factor 1 was characterised by vegetable dishes, fruit, fish and poultry and factor 2 was characterised by red or processed meat, white bread, fried fish and hot chips. Tucker’s coefficient of congruence indicated that these two dietary patterns had fair to good similarity between 2010 and 2012 (coefficient of congruence 0.96 and 0.93). While the two factors identified in 2014 were qualitatively similar to the 2010 factors, quantitatively, they were different with low coefficient of congruence i.e. the factor loadings were different between the time points, (coefficient of congruence 0.66 and 0.72). For women, PCA identified two dietary patterns at each time point. Factor 1 was characterised by vegetables, fruit and fish and factor 2 was characterised by cakes, processed meat, hot chips and confectionary (Table 2). Fair to good similarity between dietary patterns was determined by the coefficient of congruence (coefficient of congruence range 0.90 to 0.97). The factor scores of all the PCA-derived dietary patterns significantly decreased over 4 years in both men and women (Table 3). Given the equation to calculate the factor scores (the sum of the 2010 factor loadings multiplied food frequency of key food groups), this suggests that the food frequency of the food groups decreased.

**Table 2:** 2010 key food group factor loadings for the two dietary patterns derived by principal component analysis^a^

Men		Women	
2010 Factor 1		2010 Factor 1	
Eigenvalue	4.39	Eigenvalue	4.19
Variance explained	5.8%	Variance explained	7.8%
Vegetable dishes	0.31	Other vegetables	0.34
Fish and other seafood	0.31	Salad vegetables	0.34
Oil and vinegar salad dressing	0.31	Vegetable dishes	0.29
Salad vegetables	0.28	Dark green and cruciferous vegetables	0.29
Rice	0.24	Fruit	0.26
Legumes or beans	0.22	Fish and other seafood	0.25
Cottage or ricotta cheese	0.22	Orange vegetables	0.25
Fruit	0.22	Legumes or beans	0.23
Poultry	0.20	Nuts or seeds	0.23
Potato	−0.21		
2010 Factor 2		2010 Factor 2	
Eigenvalue	2.22	Eigenvalue	3.26
Variance explained	5.6%	Variance explained	6.5%
Processed or cured meat	0.29	Cakes, pastries or other desserts	0.27
Pizza and/or Hamburger	0.28	Processed or cured meat	0.26
Red meat	0.28	Sweet biscuits	0.25
White bread	0.25	Hot chips, roast potato or wedges	0.23
Fried or battered fish	0.25	Chocolate or confectionary	0.23
High-joule drinks	0.23	High-joule drinks	0.23
Hot chips, roast potato or wedges	0.20	Meat pie or sausage rolls	0.22
Muesli or porridge	−0.20	Potato	0.21
Reduced fat milk	−0.22		

^a^Only food groups with factor loadings | ≥ 0.2| are displayed in table and are listed in order for simplicity and ease of interpretation

**Table 3:** Mean factor scores and mixed-effect multilinear regression (MLR) coefficient to assess change in dietary pattern scores

Men					
	2010	2012	2014	Mixed-effect MLR	
	mean (SD)^a^			β (95% CI)	P-trend
Factor 1 Vegetable dishes, fruit, fish & poultry	1.46 (0.78)	1.32 (0.70)	1.32 (0.73)	−0.07 (− 0.08, − 0.05)	< 0.001
Factor 2 Red or processed meat, white bread, fried fish & hot chips	0.48 (0.54)	0.43 (0.48)	0.39 (0.45)	−0.04 (− 0.05, − 0.03)	< 0.001
DGI-2013 score	82.2 (14.2)	82.9 (14.1)	83.0 (14.1)	0.42 (0.16, 0.69)	0.002
Women					
	2010	2012	2014	Mixed-effect MLR	
	mean (SD)^a^			β (95% CI)	P-trend
Factor 1 Vegetables, fruit & fish	1.54 (0.74)	1.51 (0.72)	1.48 (0.70)	−0.03 (−0.04, −0.01)	*P* < 0.001
Factor 2 Cakes, processed meat, hot chips & confectionary	0.24 (0.22)	0.22 (0.20)	0.21 (0.18)	−0.01 (− 0.02, − 0.01)	*P* < 0.001
DGI-2013 score	90.4 (13.4)	90.6 (13.1)	90.6 (13.1)	0.07 (−0.19, 0.33)	0.584

Abbreviation: *MLR* mixed-effect multilinear regression *SD* standard deviation *CI* Confident interval

^a^ Values are mean factor scores calculated using baseline (2010) factor loadings at all time points

#### Diet quality

Over 4 years, the total DGI-2013 score increased in men (β = 0.42, 95%CI 0.16, 0.69, *P* = 0.002) while for women it did not significantly change (β = 0.07, 95%CI -0.19, 0.33, *P* = 0.584) (Table 3). Examining individual components of the DGI (Additional file 4) demonstrated that compliance to the diet variety and water intake recommendations decreased in men and women. Compliance with recommendations for vegetables, salt, sugar and alcohol intake increased in men over the 4 years. For women, compliance with fruit, cereal and saturated fat recommendations decreased, while compliance to discretionary food and sugar intake recommendations increased.

### Predictors of change in dietary patterns

#### PCA-derived dietary patterns

For men, having a higher level of education, trying to avoid gaining weight in 2010 and meeting physical activity recommendations in 2010 were predictors of an increase in factor 1 score (vegetable dishes, fruit, fish and poultry) by 2014 (β = 0.14: 95% CI: 0.06, 0.22: *P* = 0.001; β = 0.08: 0.01, 0.15: *P* = 0.023 and β = 0.09: 0.02, 0.16: *P* = 0.008, respectively) (Table 4). Men who reported trying to lose weight in 2010 experienced a decrease in factor 2 score (red or processed meat, white bread, fried fish and hot chips) over 4 years, compared to those not trying anything for their weight (β = − 0.07: − 0.13, − 0.01: *P* = 0.018). Men who reported being smokers in 2010 experienced an increase in factor 2 (red or processed meat, white bread, fried fish and hot chips) score over the 4 years compared to those who never smoked (β = 0.12: 0.04, 0.20: *P* = 0.004). Among women, while a number of variables were associated with changes in dietary patterns (trying to lose weight, meeting physical activity recommendations and education), there were no significant findings in women after adjusting for covariates (Table 5).

**Table 4:** Multiple linear regression of predictors of dietary change in principal component scores over four-years in men^a^

		Factor 1 Vegetable dishes, fruit, fish & poultry								Factor 2 Red or processed meat, white bread, fried fish & hot chips							
	Model 1^b^					Model 2^c^				Model 1				Model 2			
	n	β	95% CI		*P*-Value	β	95% CI		*P*-Value	β	95% CI		P-Value	β	95% CI		P-Value
Up to year 10	239	Ref.	–	–	–	Ref.	–	–	–	Ref.	–	–	–	Ref.	–	–	–
Year 12, trade or certificate	393	0.05	−0.02	0.13	0.150	0.05	− 0.02	0.12	0.172	−0.002	− 0.06	0.06	0.952	−0.001	− 0.06	0.06	0.970
University degree	319	0.17	0.09	0.24	< 0.001	0.14	0.06	0.22	0.001	−0.04	−0.11	0.02	0.195	−0.04	−0.11	0.03	0.277
Retired	329	0.03	−0.04	0.10	0.401	0.03	−0.05	0.10	0.495	−0.006	−0.05	0.04	0.790	0.02	−0.06	0.11	0.600
Relationship status																	
Living as married	851	Ref.	–	–	–	Ref.	–	–	–	Ref.	–	–	–	Ref.	–	–	–
Separated, divorced or widowed	99	−0.01	− 0.11	0.08	0.796	−0.003	− 0.10	0.09	0.949	−0.04	−0.14	0.07	0.530	−0.04	−0.14	0.06	0.443
Never married	55	0.02	−0.13	0.17	0.806	0.04	−0.12	0.19	0.638	0.007	−0.09	0.10	0.881	0.006	−0.10	0.09	0.906
Weight loss intentions																	
Not trying anything for my weight	480	Ref.	–	–	–	Ref.	–	–	–	Ref.	–	–	–	Ref.	–	–	–
Trying to avoid gaining weight	265	0.09	0.02	0.15	0.008	0.08	0.01	0.15	0.023	−0.06	− 0.12	− 0.0006	0.048	− 0.05	− 0.11	− 0.007	0.083
Trying to lose weight	260	0.02	−0.07	0.11	0.666	0.03	−0.07	0.13	0.502	−0.07	−0.13	− 0.02	0.010	− 0.07	−0.13	− 0.01	0.018
Smoking status																	
Never smoked	461	Ref.	–	–	–	Ref.	–	–	–	Ref.	–	–	–	Ref.	–	–	–
Former smoker	432	−0.04	− 0.10	0.01	0.112	− 0.08	− 0.16	0.007	0.074	−0.007	−0.07	0.05	0.805	−0.009	− 0.07	0.05	0.775
Current smoker	112	−0.09	− 0.21	0.03	0.154	−0.10	− 0.26	0.07	0.235	0.12	0.04	0.19	0.003	0.12	0.04	0.20	0.004
Meeting PA guidelines	525	0.11	0.04	0.17	0.003	0.09	0.02	0.16	0.008	−0.02	−0.06	0.03	0.477	− 0.007	− 0.05	0.04	0.745
Diagnosis of CM-related condition																	
*No CM-related conditions*	*384*	*Ref.*	*–*	*–*	*–*	*Ref.*	*–*	*–*	*–*	*Ref.*	*–*	*–*	*–*	*Ref.*	*–*	*–*	*–*
Diagnosed prior to 2010	491	0.03	−0.04	0.10	0.352	0.06	−0.01	0.14	0.111	0.02	−0.03	0.06	0.528	0.02	−0.03	0.06	0.466
Newly diagnosed within 2010–2014	130	0.04	−0.10	0.18	0.593	0.06	−0.08	0.20	0.385	−0.07	−0.15	− 0.01	0.101	− 0.07	− 0.15	0.02	0.102

Abbreviations: *CI* confidence interval. *Ref.* reference category, *PA* physical activity, *CM* cardiometabolic

^a.^ Baseline (2010) characteristics unless otherwise indicated

^b.^ Model 1: adjusted for sampling postcode clustering and baseline PCA score

^c.^ Model 2: adjusted for model 1 and additionally adjusted for age, body mass index, smoking and physical activity, where appropriate

**Table 5:** Multiple linear regression of predictors of dietary change in principal component scores at four-years in women^a^

		Factor 1 Vegetables, fruit, & fish								Factor 2 Cakes, processed meat, hot chips & confectionary							
	Model 1^b^					Model 2^c^				Model 1^b^				Model 2			
	n	β	95% CI		*P*-Value	β	95% CI		*P*-Value	β	95% CI		*P*-Value	β	95% CI		*P*-Value
Education																	
up to year 10	400	Ref.	–	–	–	Ref.	–	–	–	Ref.	–	–	–	Ref.	–	–	–
Year 12, trade or certificate	353	0.03	−0.06	0.11	0.517	0.03	−0.05	0.11	0.489	−0.01	−0.03	0.009	0.248	−0.01	−0.03	0.01	0.314
University degree	352	0.05	−0.04	0.13	0.277	0.04	−0.05	0.12	0.403	−0.02	−0.05	− 0.003	0.029	− 0.02	−0.04	0.001	0.065
Retired	499	− 0.03	−0.09	0.03	0.315	− 0.05	−0.11	0.008	0.091	0.001	−0.02	0.02	0.893	−0.007	−0.03	0.01	0.499
Relationship status																	
Living as married	854	Ref.	–	–	–	Ref.	–	–	–	Ref.	–	–	–	Ref.	–	–	–
Separated, divorced or widowed	208	−0.01	−0.10	0.07	0.774	−0.002	− 0.09	0.09	0.968	−0.04	−0.04	0.01	0.289	−0.02	−0.04	0.006	0.126
Never married	43	− 0.02	−0.19	0.14	0.765	−0.02	−0.18	0.15	0.830	−0.007	−0.06	0.05	0.797	0.006	−0.06	0.05	0.815
Weight loss intentions																	
Not trying anything for my weight	480	Ref.	–	–	–	Ref.	–	–	–	Ref.	–	–	–	Ref.	–	–	–
Trying to avoid gaining weight	265	−0.01	− 0.08	0.06	0.788	−0.02	− 0.09	− 0.17	0.577	− 0.01	− 0.03	0.003	0.093	− 0.01	− 0.03	0.005	0.185
Trying to lose weight	260	−0.09	−0.17	− 0.02	0.017	− 0.08	−0.16	0.006	0.067	0.009	−0.03	0.01	0.418	−0.004	−0.03	0.02	0.746
Smoking status																	
Never smoked	640	Ref.	–	–	–	Ref.	–	–	–	Ref.	–	–	–	Ref.	–	–	–
Former smoker	373	−0.06	−0.13	0.02	0.121	−0.06	−0.13	0.02	0.147	−0.01	−0.03	0.008	0.242	−0.01	−0.03	0.009	0.301
Current smoker	92	−0.11	−0.24	0.02	0.108	−0.10	−0.24	0.01	0.102	0.03	−0.004	0.06	0.166	0.03	−0.01	0.07	0.142
Meeting PA guidelines	634	0.07	0.01	0.13	0.019	0.06	−0.0009	0.12	0.054	−0.008	−0.03	0.01	0.425	−0.006	− 0.03	0.01	0.512
Diagnosis of CM-related condition																	
No CM-related conditions	491	Ref.	–	–	–	Ref.	–	–	–	Ref.	–	–	–	Ref.	–	–	–
Diagnosed prior to 2010	493	−0.03	−0.10	0.04	0.419	−0.006	− 0.08	0.07	0.882	0.002	−0.02	0.02	0.830	−0.003	−0.02	0.01	0.769
Newly diagnosed within 2010–2014	121	−0.10	− 0.22	0.02	0.113	−0.09	−0.21	0.04	0.187	−0.02	−0.04	0.009	0.199	−0.02	−0.05	0.004	0.113

Abbreviations: *CI* confidence interval. *Ref.* reference category, *PA* physical activity. CM, cardiometabolic

^a.^ Baseline (2010) characteristics unless otherwise indicated

^b.^ Model 1: adjusted for sampling postcode clustering and baseline PCA score

^c.^ Model 2: adjusted for model 1 and additionally adjusted for age, body mass index, smoking and physical activity, where appropriate

#### Diet quality

Compared to those that reported never smoking, being a smoker in 2010 was associated with a decrease in DGI-2013 (β = − 4.77: − 6.76, − 2.78: *P* < 0.001 and β = − 4.11: − 6.92, − 1.31: *P* = 0.005 for men and women, respectively) (Table 6). A diagnosis of a cardiometabolic-related condition prior to 2010 was associated with an increase in diet quality in men over 4 years compared to not having been diagnosed (β = 1.25: 0.09, 0.58: *P* = 0.035). There were no other significant associations identified for men or women.

**Table 6:** Multiple linear regression of predictors of dietary change in diet quality at four-years in men and women^a^

	Men (*n* = 1005)									Women (*n* = 1106)								
	Model 1^b^					Model 2^c^				Model 1					Model 2			
	n	β	95% CI^c^		*P*-Value	β	95% CI		*P*-Value	n	β	95% CI		*P*-Value	β	95% CI		*P*-Value
Education																		
Up to year 10	239	Ref.	–	–	–	Ref.	–	–	–	400	Ref.	–	–	–	Ref.	–	–	–
Year 12, trade/apprenticeship or certificate/diploma	393	0.0	−1.37	1.42	0.970	−0.01	−1.36	1.34	0.988	353	0.45	−0.99	1.90	0.535	0.42	− 1.01	1.85	0.562
University degree and higher	319	1.24	−0.36	2.83	0.128	0.78	−0.85	2.42	0.334	352	−0.09	−1.53	1.35	0.902	−0.19	−1.64	1.26	0.792
Weight loss intentions																		
Not trying anything for my weight	480	Ref.	–	–	–	Ref.	–	–	–	480	Ref.	–	–	–	Ref.	–	–	–
Trying to avoid gaining weight	265	1.21	−0.15	2.56	0.080	0.95	−0.45	3.34	0.180	265	0.97	−0.39	2.34	0.160	0.87	−0.53	2.27	0.221
Trying to lose weight	260	0.86	−0.79	2.51	0.302	0.71	−1.10	2.52	0.437	260	−0.09	− 1.80	1.61	0.912	−0.02	− 1.81	1.86	0.979
Retired	329	0.58	−0.64	1.80	0.349	0.46	−0.88	1.79	0.498	499	0.17	−1.16	1.50	0.800	0.25	−1.19	1.68	0.732
Relationship status																		
Married	851	Ref.	–	–	–	Ref.	–	–	–	854	Ref.	–	–	–	Ref.	–	–	–
Separated	99	0.50	−1.39	2.40	0.598	0.70	−1.19	2.60	0.463	208	0.73	−0.89	2.35	0.201	1.20	−0.33	2.74	0.123
Never married	55	0.20	−2.03	2.43	0.857	0.26	−2.13	2.65	0.830	43	2.81	−0.49	6.10	0.094	2.86	−0.33	6.04	0.078
Smoking status																		
Non smoker	461	Ref.	–	–	–	Ref.	–	–	–	640	Ref.	–	–	–	Ref.	–	–	–
Use to smoke	432	−1.83	−3.16	−0.49	0.008	−1.87	−3.24	−0.50	0.008	373	− 1.86	−2.91	−0.74	0.001	− 1.87	− 2.99	− 0.75	0.001
Smoker	112	−4.89	−6.88	−2.90	< 0.001	−4.77	−6.76	−2.78	< 0.001	92	−4.07	−6.87	−1.28	0.005	−4.11	−6.92	−1.31	0.005
Meeting PA guidelines	525	0.89	−0.32	2.11	0.147	0.71	−0.43	1.86	0.219	634	0.47	−0.86	1.80	0.486	0.32	−1.04	1.67	0.645
Diagnosis of CM-related condition																		
No CM-related conditions	384	Ref.	–	–	–	Ref.	–	–	–	491	Ref.	–	–	–	Ref.	–	–	–
Diagnosed prior to 2010	491	1.10	−0.03	2.23	0.056	1.25	0.09	2.42	0.035	493	0.30	−1.05	1.64	0.661	0.65	−0.71	2.00	0.345
Newly diagnosed within 2010–2014	130	1.51	−0.48	3.50	0.135	1.70	−0.20	3.61	0.079	121	0.70	−1.41	2.80	0.512	0.95	−1.25	3.15	0.391

Abbreviations: *CI* confidence interval. *Ref.* reference category, *PA* physical activity, *CM* cardiometabolic

^a.^ Baseline (2010) characteristics unless otherwise indicated

^b.^ Model 1: adjusted for sampling postcode clustering and baseline PCA score

^c.^ Model 2: adjusted for model 1 and additionally adjusted for age, body mass index, smoking and physical activity, where appropriate

## Discussion

This longitudinal study adds to the limited research regarding dietary patterns over time and contributing to our understanding of the predictors of dietary patterns in adults of peri-retirement age. Qualitatively, there was stability in the main dietary patterns that were identified over time in the men and women, with a healthy and an unhealthy dietary pattern consistently identified. However, scores on these dietary patterns decreased over the 4 years in men and women, indicating that participants may have reported an overall decrease in the frequency of consumption of key food groups. Diet quality as assessed by the DGI-2013 increased from 2010 to 2014 in men, while no change was found in women, however compliance with the Australian Dietary Guidelines remained poor. Of the predictors examined, men with a higher level of education, not smoking and meeting the physical activity recommendations were predictors of a favorable change in dietary patterns .

The key dietary patterns identified remained consistent across time, however changes in consumption were observed. All the factor scores of the dietary patterns determined by PCA decreased over time suggesting that there may have been an overall decrease in the consumption of the key food items by this population. Diet quality, as assessed by the DGI-2013, increased from 2010 to 2014 in men, while no change was observed in women. Previous research has shown positive changes in diet quality scores over time [52–54]. Trends in the individual DGI-2013 components differed, with men making some favourable changes to their diet by increasing compliance with the vegetable intake guideline and decreasing their salt use, extra sugar use and alcohol consumption. However, diet variety, a favourable dietary behaviour, also decreased. Poorer dietary changes were identified in women, with a decrease in diet variety, fruit intake and water consumption and an increase in saturated fat observed, while favourable changes including reduced discretionary foods and extra sugar intake were also seen. Compliance to the overall Australian Dietary Guidelines remained low, with a mean (SD) DGI-2013 score of 83.1 (14.1) and 90.6 (13.1) for men and women, respectively in 2014, out of a total achievable score of 130.

It may be that the decrease in diet variety in men and women resulted from the overall decrease in food intake observed in the analysis of the PCA-derived dietary patterns (indicated by the negative change for all factor scores). There have been mixed results in previous studies with respect to diet variety and age. Some studies have reported increased diet variety with age [55–57], while others have shown a decrease [58]. Measurement of diet variety in the current study may be limited by the use of a FFQ to collect dietary intake information with a restricted number of food items included.

When examining predictors of dietary change, we found that higher education and other healthy lifestyle characteristics including not smoking, meeting physical activity recommendations and avoiding weight gain predicted favourable changes in dietary patterns. These results are in line with previous studies [16, 19, 59]. It is well understood that higher education is often associated with health-promoting dietary behaviours [48]. The relationship between diet and socioeconomic position is complex [60] and there is a need to consider those with low socioeconomic position when targeting nutritional messages. Smoking status and previous diagnosis of a cardiometabolic-related condition were the only factors that predicted change in diet quality. However, as there was very little change in the DGI-2013 score overall, power was limited in this analysis.

Smoking is often coupled with other negative health behaviours such as poor diet and physical inactivity and the combined effects of these risk factors lead to poor health outcomes [61]. This was supported in the current study, in that smoking predicted a decrease in diet quality over time and not meeting physical activity recommendations predicted a decrease in favourable dietary patterns. In line with this, the 1958 British Birth Cohort found those who increased their physical activity between the age of 33 and 42 years, also made improvements in diet [62].

Few studies have explored change in dietary patterns longitudinally, with most assessing diet at just one time point. Comparison of dietary patterns identified by the empirically-based techniques across different time points presents challenges. Since PCA identifies patterns within the available data, assessing the derived patterns at different time points may result in different patterns identified or factor loading of similar patterns will have unavoidably changed. For PCA, calculating PCA factor scores based on the baseline factor loadings rather than comparing the dietary patterns identified at each time point helps to overcome these concerns [18, 19]. However, this technique is limited if the overall dietary patterns are not similar across time or when comparing different population groups. In the current study, we found that the PCA-derived dietary patterns were comparable across time in terms of the type of food present. However, in men the coefficient of congruence indicated that the identified patterns were not quantitatively equal over time. This suggests that while similar food groups loaded highly on the dietary patterns identified at each time, their factor loadings were different. This is a limitation of using empirical dietary pattern methods in longitudinal studies and is why it is important to use consistent factor loadings over time.

Limited studies have explored empirically derived dietary patterns longitudinally [17, 18, 23, 53, 59, 63] and there is little consensus on the methodological approaches to use. Mishra et al. (2006) used exploratory factor analysis to determine dietary patterns at baseline, and similar to the present study, they used an equation defined at baseline to calculate the diet scores at the two follow-up times. Using this approach enables researchers to assess the changes in dietary pattern identified at baseline but does not allow for new dietary patterns that may have emerged over time [64].

Dietary patterns assessed using diet quality scores are simpler to compare over time since they are not data-driven and criteria for scoring can be applied consistently across time. However, as diet quality indices are based on prior knowledge or existing national guidelines they require significant efforts to design them and to ensure validity prior to use [65]. A potential limitation of interpreting the overall dietary patterns derived by either approach, is that they encompass multiple components of diet and it is possible that there are different predictors of change for the separate components of diet quality.

This study has a number of strengths and limitations. Strengths of this study include the population-based design of the WELL study, the focus on older adults and the comparison of different methods of assessing dietary patterns. Although the response rate was modest (38%), the sampling technique resulted in a sample with characteristics consistent with both state [66] and national data [67, 68] maximising the generalisability of the study results. Furthermore, the specific age range of 55–65 years captures an understudied population during a transitional life stage and the comparative nature of this study adds to the limited research in this area.

The use of FFQs are known to be susceptible to measurement error of dietary intake. The FFQ used in this study has previously been used to assess dietary patterns and behaviours, demonstrating that it is a valid predictor of health outcomes and suggesting it has predictive validity [7, 29, 39]. Portion sizes were not measured in this study and the FFQ assumes that each eating occasion was a standard serve of the food or food group. This is a limitation of the dietary pattern methodologies used. Further to this, energy intake could not be estimated and used to adjust for in the analyses of input variables for dietary pattern analysis and predictors of change nor could under-reporters be identified. However, research shown that frequency of intake is the major determinant of energy intake [69, 70] and non-energy adjusted frequency is more sensitive to the intake of important low-energy foods such as fruit and vegetables [71, 72].

This study relied on self-reported measures, which may result in measurement error, for example height, weight and BMI. However, self-reported height and weight has previously been shown to be a valid estimate of BMI in large epidemiological studies [73, 74]. The short follow-up period was a further limitation, which meant that few participants experienced change in predictor variables, therefore this could not be considered in the analyses.

Both empirical-based dietary pattern and theoretical techniques have inherent limitations and considerations for dietary pattern analysis. Several researcher-determined decisions are required such as determining the index components and scoring methods, the collapsing and format of input variable, the number of derived empirical patterns and assigning labels to patterns for example [37, 65, 75]. Steps were taken to reduce subjectivity. For example, the FFQ foods were grouped based on approaches used in previous literature and consistent with the Australian Dietary Guidelines [36]. Established criteria and best practice were used to determine the dietary patterns and objective criteria were used to compare the dietary patterns between men and women in PCA.

## Conclusions

In this sample of peri-retirement aged adults the dietary patterns identified over 4 years remained stable in men and women. Changes observed within individuals included reduced consumption of key foods within the PCA-determined dietary patterns in both men and women. While there were improvements in diet quality in men, overall diet quality remains poor. Several predictors of changes in dietary patterns were identified, with higher education and favourable baseline lifestyle characteristics tending to predict an increase in healthier dietary patterns. Design of nutrition promotion initiatives for older adults need to consider those with lower socioeconomic status, as having a lower level of education was a predictor of poorer dietary patterns. It is also important to consider how nutrition behaviours can be targeted alongside other lifestyle behaviours, such as smoking and inadequate physical activity recommendations to improve health. This study adds to the limited literature of longitudinal dietary pattern and dietary patterns of peri-retirement aged adults.

## Supplementary information

**Additional file 1.** The Wellbeing Eating and Exercise for a Long Life study participant recruitment flow diagram

**Additional file 2.** Factor loadings for dietary patterns derived by principal component analysis at three time points in men of the Wellbeing Eating and Exercise for a Long Life Study

**Additional file 3.** A list of the 52 food groups derived from the 111 items in the food frequency questionnaire

**Additional file 4.** Examining change in individual components of the DGI over three time points

## Data Availability

The datasets used and/or analysed during the current study are available from the corresponding author on reasonable request.

## Abbreviations

95% CI: 95% confidence interval
ADG: Australian Dietary Guidelines
BMI: Body Mass Index
CVD: Cardiovascular disease
DGI-2013: 2013 Revised Dietary Guideline Index
FFQ: Food Frequency Questionnaire
PCA: Principal component analysis
SEIFA: Index of Relative Socio-economic Disadvantage, a Socioeconomic Index for Areas score
SEP: Socioeconomic Position
WELL: Wellbeing Eating and Exercise for a Long Life study
