# Supplementary material for: Education and lifestyle predict change in dietary patterns and diet quality of adults 55 years and over
Source: Nutr J. 2019 Nov 7;18:67. doi: 10.1186/s12937-019-0495-6 (PMC6839215; doi:10.1186/s12937-019-0495-6)
Supplement: Supplementary file 1 — Additional file 1. The Wellbeing Eating and Exercise for a Long Life study participant recruitment flow diagram [file 12937_2019_495_MOESM1_ESM.docx]

Participant recruitment flow diagram of the Wellbeing Eating and Exercise for Long Life (WELL) study

T3 questionnaires sent

n=3,123

T2 questionnaires returned

n=2,757

Low socioeconomic position

14 rural post codes × n=134

14 urban post codes × n=134

Med socioeconomic position

14 rural post codes × n=134

14 urban post codes × n=134

High socioeconomic position

14 rural post codes × n=134

14 urban post codes × n=134

n=11,256

Ineligible subjects n=475

Out of 55-65 age range n=95

Invitation not delivered n=380

2010 questionnaires sent

n=10,781

2010 questionnaires returned

n=4,082

Subjects withdrew n=714

Invitation not delivered n=380

T2 questionnaires sent

n=3,368

Subjects withdrew n=112

- too busy n=18
- sick n=10
- deceased n=7
- away from home n=6
- provided no reason n=61

Lost contact with subjects n=72

Questionnaire lost in mail n=5

Unresolved n=422

T3 questionnaires returned

n=2,542

Subjects withdrew n=86

- too busy n=17
- sick n=4
- deceased n=6
- provided no reason n=62

Lost contact with subjects n=44

Questionnaire lost in mail n=3

Unresolved n=448
