# Supplementary material for: Education and lifestyle predict change in dietary patterns and diet quality of adults 55 years and over
Source: Nutr J. 2019 Nov 7;18:67. doi: 10.1186/s12937-019-0495-6 (PMC6839215; doi:10.1186/s12937-019-0495-6)
Supplement: Supplementary file 2 — Additional file 2. Factor loadings for dietary patterns derived by principal component analysis at three time points in men of the Wellbeing Eating and Exercise for a Long Life Study [file 12937_2019_495_MOESM2_ESM.docx]

| Factor loadings for dietary patterns derived by principal component analysis at three time points in men of the Wellbeing Eating and Exercise for a Long Life Study | | | | | | | | | | | | |
| --- | --- | --- | --- | --- | --- | --- | --- | --- | --- | --- | --- | --- |
|  | 2010 Factors  (n=1,888) | | | |  | 2012 Factors  (n=1,269) | | | |  | 2014 Factors  (n=1,183) | |
| Patterns (in order of variance explained) | i | ii | iii | iv |  | i | ii | iii | iv |  | i | iv |
| Eigenvalues | 4.39 | 3.22 | 2.22 | 2.01 |  | 4.14 | 3.68 | 2.17 | 1.99 |  | 4.2 | 3.28 |
| Variance explained | 5.8% | 5.7% | 5.6% | 5.6% |  | 7.0% | 5.7% | 5.3% | 5.1% |  | 7.8% | 6.6% |
| Vegetables and fruit | |  |  |  |  |  |  |  |  |  |  |  |
| Vegetable dishes | 0.31 | -0.01 | 0.03 | 0.13 |  | -0.04 | 0.32 | 0.15 | 0.00 |  | 0.32 | -0.01 |
| Dark green and cruciferous vegetables | 0.07 | -0.04 | -0.02 | 0.44 |  | -0.04 | 0.06 | 0.42 | 0.00 |  | 0.26 | 0.01 |
| Orange vegetables | -0.04 | 0.02 | -0.04 | 0.50 |  | -0.02 | -0.07 | 0.50 | 0.05 |  | 0.25 | 0.08 |
| Salad vegetables | 0.28 | 0.01 | -0.01 | 0.20 |  | -0.06 | 0.28 | 0.20 | 0.00 |  | 0.31 | -0.05 |
| Potato | -0.21 | 0.07 | 0.10 | 0.36 |  | 0.15 | -0.18 | 0.31 | -0.01 |  | 0.07 | 0.21 |
| Other vegetables | 0.13 | -0.05 | 0.03 | 0.44 |  | 0.01 | 0.12 | 0.46 | -0.05 |  | 0.31 | 0.08 |
| Legumes/beans | 0.22 | -0.01 | 0.01 | 0.06 |  | -0.05 | 0.26 | 0.02 | 0.03 |  | 0.20 | -0.09 |
| Fruit | 0.22 | 0.12 | -0.09 | 0.13 |  | -0.04 | 0.15 | 0.14 | 0.22 |  | 0.28 | -0.02 |
| Dried fruit | 0.16 | 0.17 | -0.08 | -0.02 |  | -0.03 | 0.09 | 0.02 | 0.22 |  | 0.19 | -0.07 |
| Nuts and/or seeds | 0.19 | 0.17 | -0.10 | -0.05 |  | 0.02 | 0.18 | 0.01 | 0.25 |  | 0.20 | 0.00 |
| Cereal |  |  |  |  |  |  |  |  |  |  |  |  |
| White bread | -0.10 | 0.09 | 0.25 | 0.04 |  | 0.25 | -0.07 | 0.00 | -0.09 |  | -0.09 | 0.22 |
| Wholegrain bread | 0.06 | 0.26 | -0.14 | 0.01 |  | 0.04 | 0.01 | 0.02 | 0.26 |  | 0.15 | 0.03 |
| Savoury crackers | 0.03 | 0.23 | -0.04 | 0.01 |  | 0.14 | 0.04 | -0.06 | 0.16 |  | 0.13 | 0.10 |
| Muesli or cooked porridge | 0.10 | 0.19 | -0.20 | 0.02 |  | -0.05 | 0.00 | 0.02 | 0.33 |  | 0.18 | -0.06 |
| Breakfast cereal | -0.11 | 0.22 | -0.08 | 0.09 |  | 0.09 | -0.18 | 0.02 | 0.23 |  | 0.02 | 0.10 |
| Rice | 0.24 | -0.06 | -0.02 | -0.05 |  | 0.02 | 0.27 | -0.15 | 0.04 |  | 0.14 | -0.05 |
| Pasta | 0.19 | 0.06 | 0.04 | -0.10 |  | 0.09 | 0.26 | -0.16 | 0.05 |  | 0.12 | 0.03 |
| Meat |  |  |  |  |  |  |  |  |  |  |  |  |
| Red meat | 0.03 | -0.01 | 0.28 | 0.12 |  | 0.19 | 0.08 | 0.10 | -0.18 |  | 0.05 | 0.19 |
| Processed or cured meat | 0.03 | 0.04 | 0.29 | 0.01 |  | 0.25 | 0.05 | 0.03 | -0.13 |  | 0.01 | 0.29 |
| Poultry | 0.20 | -0.02 | 0.12 | 0.01 |  | 0.10 | 0.15 | 0.01 | -0.03 |  | 0.12 | 0.10 |
| Fish and other seafood | 0.31 | -0.05 | 0.01 | 0.00 |  | -0.04 | 0.26 | 0.06 | 0.02 |  | 0.21 | -0.05 |
| Fried or battered fish | 0.15 | -0.01 | 0.25 | -0.01 |  | 0.12 | 0.10 | 0.02 | -0.13 |  | 0.04 | 0.16 |
| Eggs | 0.13 | 0.00 | 0.18 | 0.03 |  | 0.13 | 0.19 | 0.01 | -0.07 |  | 0.10 | 0.10 |
| Dairy |  |  |  |  |  |  |  |  |  |  |  |  |
| Flavoured milk drinks | -0.06 | 0.09 | 0.11 | -0.03 |  | 0.09 | -0.01 | 0.00 | -0.01 |  | 0.00 | 0.14 |
| Whole Milk | -0.10 | 0.12 | 0.13 | 0.01 |  | 0.16 | -0.06 | 0.01 | 0.01 |  | 0.00 | 0.15 |
| reduced fat milk | -0.02 | 0.19 | -0.22 | 0.02 |  | -0.02 | -0.09 | -0.01 | 0.28 |  | 0.08 | 0.01 |
| Cream | -0.02 | 0.08 | 0.09 | -0.01 |  | 0.13 | 0.02 | 0.02 | -0.04 |  | 0.00 | 0.18 |
| Ice-cream | -0.10 | 0.20 | 0.06 | 0.05 |  | 0.18 | -0.07 | 0.16 | 0.01 |  | -0.03 | 0.21 |
| Yoghurt | 0.13 | 0.20 | -0.15 | 0.00 |  | -0.01 | 0.09 | -0.02 | 0.31 |  | 0.17 | -0.08 |
| Cottage or ricotta cheese | 0.22 | 0.01 | 0.02 | -0.06 |  | 0.05 | 0.16 | -0.10 | 0.09 |  | 0.11 | -0.02 |
| Cheddar cheese | 0.06 | 0.22 | 0.08 | -0.04 |  | 0.15 | 0.11 | -0.04 | 0.05 |  | 0.09 | 0.13 |
| Other |  |  |  |  |  |  |  |  |  |  |  |  |
| Water | 0.12 | 0.08 | -0.04 | 0.05 |  | -0.02 | 0.12 | 0.04 | 0.16 |  | 0.16 | -0.05 |
| Coffee | 0.06 | -0.03 | 0.13 | -0.08 |  | 0.10 | 0.11 | -0.11 | -0.07 |  | -0.03 | 0.04 |
| Tea | -0.07 | 0.20 | -0.11 | 0.09 |  | 0.04 | -0.10 | 0.08 | 0.15 |  | 0.07 | 0.03 |
| Fruit or vegetable juice | 0.09 | 0.08 | 0.05 | 0.01 |  | 0.05 | 0.08 | -0.01 | 0.04 |  | 0.08 | 0.07 |
| High-joule drinks | -0.05 | 0.08 | 0.23 | 0.00 |  | 0.24 | -0.06 | 0.05 | -0.02 |  | -0.07 | 0.27 |
| Low-joule drink | 0.01 | 0.00 | 0.06 | -0.01 |  | 0.07 | 0.00 | -0.04 | 0.06 |  | -0.01 | 0.08 |
| Beer | -0.03 | -0.13 | 0.19 | 0.07 |  | 0.05 | 0.03 | 0.05 | -0.23 |  | -0.05 | 0.08 |
| Wine | 0.18 | -0.04 | 0.02 | -0.10 |  | -0.02 | 0.20 | -0.06 | -0.08 |  | 0.10 | -0.05 |
| Spirits and liqueurs | 0.06 | -0.02 | 0.13 | -0.07 |  | 0.06 | 0.09 | 0.00 | -0.07 |  | 0.02 | 0.00 |
| Cakes, pastries or desserts | -0.02 | 0.27 | 0.09 | -0.01 |  | 0.21 | 0.00 | 0.00 | 0.18 |  | 0.07 | 0.25 |
| Sweet biscuits | -0.03 | 0.28 | 0.10 | -0.08 |  | 0.30 | -0.05 | -0.07 | 0.18 |  | 0.00 | 0.27 |
| Chocolate or confectionary | 0.00 | 0.23 | 0.16 | -0.08 |  | 0.28 | 0.05 | -0.05 | 0.11 |  | 0.08 | 0.22 |
| Meat pie or sausage rolls | -0.05 | 0.02 | 0.17 | -0.01 |  | 0.27 | -0.06 | -0.04 | -0.01 |  | -0.06 | 0.17 |
| Pizza and/or Hamburger | 0.07 | -0.02 | 0.28 | -0.06 |  | 0.23 | 0.07 | -0.09 | -0.08 |  | -0.02 | 0.19 |
| Spreads and preserves | -0.01 | 0.34 | 0.00 | 0.00 |  | 0.18 | -0.05 | 0.03 | 0.26 |  | 0.11 | 0.15 |
| Potato chips etc | 0.02 | 0.08 | 0.19 | -0.07 |  | 0.22 | 0.05 | -0.07 | -0.06 |  | -0.05 | 0.19 |
| Oil and vinegar salad dressing | 0.31 | 0.01 | 0.08 | -0.05 |  | 0.02 | 0.32 | -0.02 | -0.01 |  | 0.18 | -0.05 |
| Creamy salad dressing | 0.09 | 0.08 | 0.13 | 0.02 |  | 0.12 | 0.13 | 0.07 | -0.02 |  | 0.12 | 0.09 |
| Margarine | -0.11 | 0.24 | 0.08 | 0.00 |  | 0.19 | -0.10 | 0.04 | 0.11 |  | 0.03 | 0.17 |
| Butter | 0.00 | 0.15 | 0.15 | -0.04 |  | 0.12 | 0.00 | 0.14 | -0.05 |  | 0.01 | 0.20 |
| Hot chips, roast potato or wedges | -0.10 | 0.00 | 0.20 | 0.20 |  | 0.24 | -0.03 | 0.06 | -0.13 |  | -0.08 | 0.25 |

| Factor loadings for dietary patterns derived by principal component analysis at three time points in women of the Wellbeing Eating and Exercise for a Long Life Study | | | | | | | | |
| --- | --- | --- | --- | --- | --- | --- | --- | --- |
|  | 2010 Factors  (n=2,071) | |  | 2012 Factors  (n=1,428) | |  | 2014 Factors  (n=1,309) | |
|  | i | ii |  | i | ii |  | i | ii |
| Eigenvalues | 4.19 | 3.26 |  | 4.22 | 3.33 |  | 3.93 | 3.19 |
| Variance explained | 7.8% | 6.5% |  | 8.1% | 6.4% |  | 7.6% | 6.2% |
| Vegetables and fruit | |  |  |  |  |  |  |  |
| Vegetable dishes | 0.29 | -0.05 |  | 0.32 | -0.05 |  | 0.33 | -0.02 |
| Other vegetables | 0.34 | 0.06 |  | 0.34 | 0.05 |  | 0.36 | 0.07 |
| Salad vegetables | 0.34 | -0.03 |  | 0.32 | -0.04 |  | 0.33 | -0.05 |
| Dark green and cruciferous vegetables | 0.29 | 0.04 |  | 0.29 | 0.01 |  | 0.31 | 0.04 |
| Orange vegetables | 0.25 | 0.16 |  | 0.27 | 0.07 |  | 0.27 | 0.12 |
| Potato | -0.01 | 0.21 |  | -0.02 | 0.22 |  | 0.00 | 0.28 |
| Legumes/beans | 0.23 | -0.06 |  | 0.21 | -0.10 |  | 0.22 | -0.08 |
| Fruit | 0.26 | -0.05 |  | 0.27 | -0.03 |  | 0.28 | -0.05 |
| Dried fruit | 0.15 | -0.06 |  | 0.16 | -0.02 |  | 0.13 | 0.01 |
| Nuts and/or seeds | 0.23 | -0.02 |  | 0.24 | -0.01 |  | 0.22 | -0.08 |
| Cereal |  |  |  |  |  |  |  |  |
| White bread | -0.12 | 0.18 |  | -0.04 | 0.23 |  | -0.07 | 0.18 |
| Wholegrain bread | 0.11 | 0.10 |  | 0.12 | 0.11 |  | 0.10 | 0.10 |
| Savoury crackers | 0.07 | 0.17 |  | 0.04 | 0.14 |  | 0.07 | 0.10 |
| Muesli or cooked porridge | 0.17 | -0.09 |  | 0.14 | -0.07 |  | 0.12 | -0.07 |
| Breakfast cereal | 0.04 | 0.14 |  | 0.04 | 0.15 |  | 0.00 | 0.07 |
| Rice | 0.12 | -0.02 |  | 0.12 | 0.01 |  | 0.06 | -0.01 |
| Pasta | 0.11 | 0.15 |  | 0.12 | 0.09 |  | 0.09 | 0.10 |
| Meat |  |  |  |  |  |  |  |  |
| Red meat | 0.03 | 0.19 |  | 0.10 | 0.17 |  | 0.08 | 0.25 |
| Processed or cured meat | 0.01 | 0.26 |  | 0.02 | 0.25 |  | 0.00 | 0.29 |
| Poultry | 0.09 | 0.06 |  | 0.12 | 0.06 |  | 0.14 | 0.08 |
| Fish and other seafood | 0.25 | 0.04 |  | 0.20 | -0.05 |  | 0.21 | -0.05 |
| Fried or battered fish | 0.05 | 0.12 |  | 0.02 | 0.16 |  | 0.02 | 0.14 |
| Eggs | 0.15 | 0.17 |  | 0.15 | 0.08 |  | 0.13 | 0.01 |
| Dairy |  |  |  |  |  |  |  |  |
| Flavoured milk drinks | 0.00 | 0.11 |  | 0.00 | 0.05 |  | -0.03 | 0.05 |
| Whole Milk | -0.04 | 0.09 |  | 0.04 | 0.10 |  | -0.02 | 0.02 |
| reduced fat milk | 0.06 | 0.03 |  | 0.05 | 0.02 |  | 0.03 | 0.01 |
| Cream | 0.00 | 0.11 |  | 0.00 | 0.11 |  | -0.02 | 0.20 |
| Ice-cream | 0.03 | 0.18 |  | -0.01 | 0.19 |  | 0.03 | 0.22 |
| Yoghurt | 0.16 | -0.08 |  | 0.15 | -0.03 |  | 0.14 | -0.08 |
| Cottage or ricotta cheese | 0.17 | -0.07 |  | 0.15 | -0.06 |  | 0.12 | -0.05 |
| Cheddar cheese | 0.05 | 0.15 |  | 0.11 | 0.19 |  | 0.06 | 0.11 |
| Other |  |  |  |  |  |  |  |  |
| Water | 0.15 | -0.04 |  | 0.16 | -0.03 |  | 0.16 | -0.05 |
| Coffee | 0.00 | 0.05 |  | -0.03 | 0.03 |  | -0.01 | 0.04 |
| Tea | 0.02 | 0.07 |  | 0.05 | 0.06 |  | 0.05 | 0.06 |
| Fruit or vegetable juice | 0.04 | 0.05 |  | 0.01 | 0.07 |  | 0.02 | 0.08 |
| High-joule drinks | -0.03 | 0.23 |  | -0.06 | 0.19 |  | -0.05 | 0.18 |
| Low-joule drink | -0.02 | 0.11 |  | -0.04 | 0.10 |  | -0.03 | 0.08 |
| Beer | -0.02 | 0.00 |  | -0.01 | 0.00 |  | 0.06 | 0.05 |
| Wine | 0.03 | 0.01 |  | 0.02 | -0.01 |  | 0.03 | -0.01 |
| Spirits and liqueurs | -0.02 | 0.05 |  | -0.03 | 0.04 |  | -0.05 | 0.04 |
| Cakes, pastries or desserts | 0.02 | 0.27 |  | 0.02 | 0.26 |  | 0.00 | 0.25 |
| Sweet biscuits | -0.02 | 0.25 |  | -0.03 | 0.26 |  | -0.04 | 0.20 |
| Chocolate or confectionary | 0.00 | 0.23 |  | -0.02 | 0.17 |  | -0.04 | 0.15 |
| Meat pie or sausage rolls | -0.08 | 0.22 |  | -0.07 | 0.28 |  | -0.05 | 0.29 |
| Pizza and/or Hamburger | -0.02 | 0.17 |  | 0.02 | 0.17 |  | 0.00 | 0.09 |
| Spreads and preserves | 0.03 | 0.18 |  | 0.05 | 0.24 |  | 0.07 | 0.18 |
| Potato chips etc | -0.03 | 0.16 |  | -0.04 | 0.20 |  | 0.01 | 0.20 |
| Oil and vinegar salad dressing | 0.18 | -0.03 |  | 0.22 | -0.02 |  | 0.23 | -0.03 |
| Creamy salad dressing | 0.09 | 0.14 |  | 0.11 | 0.15 |  | 0.10 | 0.17 |
| Margarine | -0.06 | 0.20 |  | -0.01 | 0.25 |  | -0.02 | 0.20 |
| Butter | -0.02 | 0.15 |  | 0.01 | 0.16 |  | 0.03 | 0.16 |
| Hot chips, roast potato or wedges | -0.01 | 0.23 |  | -0.05 | 0.20 |  | -0.01 | 0.28 |
