# Supplementary material for: Education and lifestyle predict change in dietary patterns and diet quality of adults 55 years and over
Source: Nutr J. 2019 Nov 7;18:67. doi: 10.1186/s12937-019-0495-6 (PMC6839215; doi:10.1186/s12937-019-0495-6)
Supplement: Supplementary file 3 — Additional file 3. A list of the 52 food groups derived from the 111 items in the food frequency questionnaire [file 12937_2019_495_MOESM3_ESM.docx]

| List of the 52 food groups derived from the 111 items in the food frequency questionnaire | |
| --- | --- |
| Food groups | Food items |
| Vegetables and fruit |  |
| Vegetable dishes | Green/mixed salad (including lettuce, tomato etc) in a sandwich; as a side salad/with a main meal; stir-fried or mixed vegetables; vegetable casserole |
| Dark green and cruciferous vegetables | Silverbeet or spinach; broccoli; cauliflower; brussels sprout, cabbage or coleslaw |
| Orange vegetables | Pumpkin; sweet potato; carrots |
| Salad vegetables | Capsicum; celery or cucumber; tomato; lettuce |
| Potato | Potato, boiled, mashed or baked |
| Other vegetables | Peas; green beans; zucchini, eggplant or squash; mushrooms; onion or leeks; sweetcorn |
| Legumes or beans | Soybeans or tofu; baked beans; other beans (e.g. chichpeas), lentils |
| Fruit | Apple or pear; orange, mandarin or grapefruit; bananas; peach or nectarine; plum or apricot; mango or paw paw; pineapple; grapes; melons; strawberries or other berries |
| Dried fruit | Dried fruit |
| Nuts or seeds | Peanuts, peanut butter, other nut spreads; other nuts (e.g. almonds, walnuts); seeds (e.g. sunflower, tahini) |
| Cereal |  |
| White bread | White breads, toast or rolls; English muffin, bagel or crumpet |
| Wholegrain bread | Wholemeal, mixed grain bread, toast or roll |
| Savoury crackers | Dry or savoury biscuits, crisp-bread, crackers |
| Muesli or porridge | Muesli, cooked porridge |
| Breakfast cereal | Breakfast cereal |
| Rice | Rice (white or brown) |
| Pasta | Pasta (including filed), noodles |
| Meat |  |
| Red meat | Beef, veal (roast, chop or steak); lamb (roast, chop or steak); pork (roast, chop or steak)^1^, mince dishes (e.g. rissoles, meatloaf); mixed dishes with beef, veal, lamb, pork (e.g. casserole, stir-fry); mixed dishes with pork (e.g. casserole, stir-fry); liver (including pate); other offal (e.g. kidneys) |
| Processed or cured meat | Sausage, frankfurter; bacon; ham; luncheon meats, salami |
| Poultry | Chicken, turkey, duck (roast, steamed, bbq), mixed dishes with chicken, turkey, duck (e.g. casserole, stir-fry) |
| Fish and other seafood | Canned fish (e.g. tuna, salmon, sardines); fish (steamed, baked, grilled); other seafood (e.g. prawns, squid) |
| Fried or battered fish | Fried or battered fish |
| Eggs | Eggs |
| Dairy |  |
| Flavoured milk drinks | Flavoured milk drinks (e.g. milkshakes, iced coffee, hot chocolate) |
| Whole milk | Milk as drink; milk on breakfast cereals; milk in hot beverage^2^ |
| Reduced fat milk | Milk as drink; milk on breakfast cereals; milk in hot beverage^2^ |
| Cream | Cream or sour cream |
| Ice-cream | Ice-cream |
| Yoghurt | Yoghurt (plain or flavoured) |
| Cottage or ricotta cheese | Cottage or ricotta cheese |
| Cheddar cheese | Cheddar and other cheeses |
| **Other** |  |
| Water | Water |
| Coffee | Coffee |
| Tea | Tea |
| Fruit or vegetable juice | Fruit juice (100% juice); vegetable, tomato juice |
| High-joule drinks | Fruit juice drink or fruit drink; cordial; soft drink (including flavoured mineral water) |
| Low-joule drink | Low-joule cordial; low-joule soft drink |
| Beer | Beer (low alcohol); beer (ordinary) |
| Wine | White wine or champagne, sparkling wine; red wine; wine cooler |
| Spirits and liqueurs | Sherry, port, fortified wines; spirits, liqueurs |
| Sweet biscuits | Plain sweet biscuits; cream, chocolate biscuits |
| Chocolate or confectionary | Chocolate (including chocolate bars e.g. Mars bars); other confectionary (e.g. Sweets or lollies) |
| Meat pie or sausage rolls | Meat pies, sausage roll or other savoury pastries |
| Pizza and/or hamburger | Pizza; hamburger |
| Spreads and preserves | Jam, marmalade, honey or syrups; Vegemite, Marmite or Promite |
| Potato chips etc | Potato chips, corn chips, Twisties |
| Oil and vinegar salad dressing | Oil and vinegar salad dressing |
| Creamy salad dressing | Mayonnaise or other creamy dressing |
| Margarine | Margarine on bread or cooked vegetables |
| Butter | Butter on bread or cooked vegetables |
| Hot chips or roast potato | Hot chips, roast potato or potato wedges |
| 1. Although pork is not classed as red meat for marketing purposes in Australia, the Australian Dietary Guidelines treat pork as red meat since pork is classified as red meat in the international literature, therefore for the purpose of this work pork will be classed as red meat. 2. To differentiate whole fat milk and reduced fat milk participants indicated the type of milk that they usually drink in an additional behaviour question. | |
