# Supplementary material for: Education and lifestyle predict change in dietary patterns and diet quality of adults 55 years and over
Source: Nutr J. 2019 Nov 7;18:67. doi: 10.1186/s12937-019-0495-6 (PMC6839215; doi:10.1186/s12937-019-0495-6)
Supplement: Supplementary file 4 — Additional file 4. Examining change in individual components of the DGI over three time points [file 12937_2019_495_MOESM4_ESM.docx]

| Mean 2013 revised Dietary Guideline Index scores at three time points and mixed-effect multilinear regression coefficient to assess change in scores between baseline and four-years, Wellbeing Eating and Exercise for a Long Life study | | | | | | | | | | | | | | | | | |
| --- | --- | --- | --- | --- | --- | --- | --- | --- | --- | --- | --- | --- | --- | --- | --- | --- | --- |
|  | Men (n=1,005) | | | | | | | |  | Women (n=1,106) | | | | | | | |
|  | mean ± standard deviation | | |  | Mixed-effect multilinear regression | | | |  | mean ± standard deviation | | |  | Mixed-effect multilinear regression | | | |
|  | 2010 | 2012 | 2014 |  | β | 95% CI | | P-value |  | 2010 | 2012 | 2014 |  | β | 95% CI | | P-value |
| 1. Diet variety | 4.1 ± 1.3 | 4.1 ± 1.3 | 4.1 ± 1.4 |  | -0.04 | -0.06 | -0.01 | 0.002 |  | 4.7 ± 1.4 | 4.6 ± 1.3 | 4.5 ± 1.4 |  | -0.07 | -0.10 | -0.05 | <0.001 |
| 2. Vegetables | 4.5 ± 2.5 | 4.7 ± 2.4 | 4.6 ± 2.5 |  | 0.06 | 0.008 | 0.12 | 0.025 |  | 5.9 ± 2.5 | 6.2 ± 2.4 | 6.0 ± 2.5 |  | 0.04 | -0.01 | 0.10 | 0.136 |
| 3. Fruit | 7.1 ± 3.2 | 7.0 ± 3.2 | 7.2 ± 3.2 |  | 0.03 | -0.04 | 0.10 | 0.377 |  | 8.4 ± 2.7 | 8.4 ± 2.7 | 8.2 ± 2.8 |  | -0.08 | -0.14 | -0.03 | 0.005 |
| 4. Cereal | 4.6 ± 2.1 | 4.5 ± 2.1 | 4.5 ± 2.0 |  | -0.03 | -0.08 | 0.02 | 0.220 |  | 5.5 ± 2.1 | 5.4 ± 2.1 | 5.3 ± 2.1 |  | -0.09 | -0.14 | -0.04 | <0.001 |
| 5. Lean meat | 7.9 ± 1.5 | 7.9 ± 1.5 | 7.9 ± 1.5 |  | 0.005 | -0.03 | 0.04 | 0.771 |  | 8.7 ± 1.3 | 8.7 ± 1.3 | 8.7 ± 1.3 |  | 0.006 | -0.02 | 0.04 | 0.667 |
| 6. Dairy | 5.8 ± 3.0 | 5.9 ± 3.0 | 5.9 ± 2.9 |  | 0.02 | -0.04 | 0.09 | 0.449 |  | 4.3 ± 2.5 | 4.3 ± 2.5 | 4.2 ± 2.5 |  | -0.04 | -0.09 | 0.01 | 0.138 |
| 7. Water | 6.4 ± 2.4 | 6.3 ± 2.5 | 6.3 ± 2.5 |  | -0.08 | -0.34 | -0.02 | 0.013 |  | 8.0 ± 2.2 | 7.8 ± 2.3 | 7.7 ± 2.4 |  | -0.12 | -0.18 | -0.07 | <0.001 |
| 8. Discretionary | 2.5 ± 4.3 | 2.6 ± 4.4 | 2.7 ± 4.4 |  | 0.11 | -0.003 | 0.22 | 0.056 |  | 3.5 ± 4.8 | 3.9 ± 4.9 | 4.1 ± 4.9 |  | 0.30 | 0.19 | 0.42 | <0.001 |
| 9. Saturated fat | 7.4 ± 3.0 | 7.5 ± 3.0 | 7.4 ± 3.0 |  | 0.02 | -0.04 | 0.08 | 0.488 |  | 8.7 ± 2.3 | 8.7 ± 2.3 | 8.5 ± 2.4 |  | -0.10 | -0.15 | -0.05 | <0.001 |
| 10. Unsat fat | 9.8 ± 1.5 | 9.6 ± 2.0 | 9.7 ± 1.7 |  | -0.03 | -0.08 | 0.02 | 0.285 |  | 8.4 ± 3.7 | 8.0 ± 4.0 | 8.5 ± 3.6 |  | 0.05 | -0.05 | 0.16 | 0.304 |
| 11. Added salt | 6.1 ± 3.3 | 6.2 ± 3.3 | 6.3 ± 3.2 |  | 0.08 | 0.03 | 0.13 | 0.002 |  | 6.9 ± 3.1 | 6.9 ± 3.0 | 6.8 ± 3.0 |  | -0.04 | -0.09 | 0.01 | 0.130 |
| 12. Extra sugar | 7.7 ± 4.2 | 8.1 ± 3.9 | 8.0 ± 4.0 |  | 0.14 | 0.04 | 0.25 | 0.007 |  | 8.1 ± 4.0 | 8.3 ± 3.8 | 8.4 ± 3.7 |  | 0.18 | 0.08 | 0.28 | 0.001 |
| 13. Alcohol | 8.4 ± 3.7 | 8.6 ± 3.5 | 8.6 ± 3.5 |  | 0.11 | 0.03 | 0.20 | 0.011 |  | 9.5 ± 2.2 | 9.5 ± 2.1 | 9.5 ± 2.1 |  | 0.04 | -0.02 | 0.10 | 0.156 |
| Total DGI-2013 | 82.2±14.2 | 82.9±14.1 | 83.0±14.1 |  | 0.42 | 0.16 | 0.69 | 0.002 |  | 90.4±13.4 | 90.6±13.1 | 90.6±13.1 |  | 0.07 | -0.19 | 0.33 | 0.584 |
|  | | | | | | | | | | | | | | | | | |
